# Two dynamic regimes in the human gut microbiome

**DOI:** 10.1371/journal.pcbi.1005364

**Published:** 2017-02-21

**Authors:** Sean M. Gibbons, Sean M. Kearney, Chris S. Smillie, Eric J. Alm

**Affiliations:** 1 Department of Biological Engineering, Massachusetts Institute of Technology, Cambridge, MA, United States of America; 2 The Broad Institute, Cambridge, MA, United States of America; 3 The Center for Microbiome Informatics and Therapeutics, Cambridge, MA, United States of America; Universtiy of Washington, UNITED STATES

## Abstract

The gut microbiome is a dynamic system that changes with host development, health, behavior, diet, and microbe-microbe interactions. Prior work on gut microbial time series has largely focused on autoregressive models (e.g. Lotka-Volterra). However, we show that most of the variance in microbial time series is non-autoregressive. In addition, we show how community state-clustering is flawed when it comes to characterizing within-host dynamics and that more continuous methods are required. Most organisms exhibited stable, mean-reverting behavior suggestive of fixed carrying capacities and abundant taxa were largely shared across individuals. This mean-reverting behavior allowed us to apply sparse vector autoregression (sVAR)—a multivariate method developed for econometrics—to model the autoregressive component of gut community dynamics. We find a strong phylogenetic signal in the non-autoregressive co-variance from our sVAR model residuals, which suggests niche filtering. We show how changes in diet are also non-autoregressive and that Operational Taxonomic Units strongly correlated with dietary variables have much less of an autoregressive component to their variance, which suggests that diet is a major driver of microbial dynamics. Autoregressive variance appears to be driven by multi-day recovery from frequent facultative anaerobe blooms, which may be driven by fluctuations in luminal redox. Overall, we identify two dynamic regimes within the human gut microbiota: one likely driven by external environmental fluctuations, and the other by internal processes.

## Author summary

Dynamics reveal crucial information about how a system functions. In this study, we develop an approach for disentangling two types of dynamics within the human gut microbiome. We find that autoregressive dynamics involve recovery from large deviations in community structure. These recovery processes appear to involve the blooming of facultative anaerobes and aerotolerant taxa, likely due to transient shifts in redox potential, followed by re-establishment of obligate anaerobes. Non-autoregressive dynamics carry a strong phylogenetic signal, wherein highly related taxa fluctuate coherently. These non-autoregressive dynamics appear to be driven by external, non-autoregressive variables like diet. We find that most of the community variance is driven by day-to-day fluctuations in the environment, with occasional autoregressive dynamics as the system recovers from larger shocks. Despite frequently observed disruptions to the gut ecosystem, there exists a returning force that continually pushes the gut microbiome back towards its steady-state configuration.

## Introduction

### The dynamic microbiome

Microbial ecology has become an important branch of medical science [1]. Recent work has shown how each person maintains a fairly unique microbial fingerprint [2–4], and that microbial dysbioses are often associated with shifts in health-status [5–8]. We now recognize that our microbiota are highly dynamic, and that these dynamics are linked to ecological resilience and host health [9–11]. The field has not yet settled upon whether gut microbial community structure varies continuously or if it jumps between discrete community states, and whether these states are shared across individuals [12–14]. In particular, some researchers suggest that gut communities can be binned into discrete ‘enterotypes’ [12], while others argue that gut communities vary along multidimensional continua [13]. If the ultimate goal of microbiome research is to improve human health by engineering the ecology of the gut, we must first understand how and why our microbiota vary in time, whether these dynamics are consistent across humans, and whether we can define ‘stable’ or ‘healthy’ dynamics.

Gut microbiota are continually buffeted by external factors like diet and host behavior [10, 15]. Internal species-species (e.g. cross-feeding or successional turnover) and host-species (e.g. immune system regulation or host physiology) interactions can also influence the structure and stability of the gut ecosystem [16–18]. Lotka-Volterra (LV) models, which predict changes in community composition through defined species-species or species-resource interaction terms, are popular for describing these internal ecological dynamics [19–22]. LV models are deterministic and fairly straight-forward to interpret, but little is known about the relative importance of these purely autoregressive factors in driving gut microbial dynamics (see *Theoretical Considerations* section below for a more detailed comparison of LV and VAR models). More recently, a model-free approach to forecasting non-linear dynamics—called convergent cross-mapping (CCM)—has been applied to ecological time series data [23]. While extremely useful, CCM can be difficult to interpret [24, 25], and may not be appropriate for high-dimensional systems with weak coupling between components (e.g. the gut).

Long, dense time series data are becoming increasingly available to microbial ecologists [10, 11, 26]. These temporal data are invaluable for understanding the behavior of microbial communities but require special care during analysis due to the non-independence of temporally adjacent samples [27]. In this paper we conduct a meta-analysis of the four longest, densest human gut time series currently available [10, 11].

### Time series analysis approach

**Fig 1:**
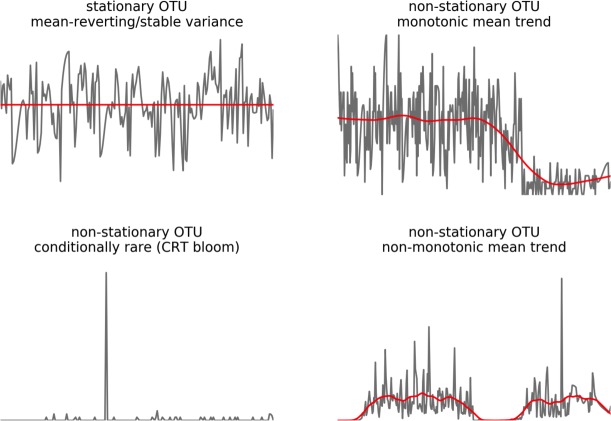
Time series stationarity and non-stationarity. Grey lines depict time series, with the mean plotted in red. The top left time series is stationary, with a stable mean (i.e. mean-reverting) and variance (i.e. non-heteroskedastic). The top right trace shows a non-stationary time series where the mean and variance change monotonically. The bottom left trace shows a time series with non-stationary variance (i.e. a sudden bloom event). The bottom right trace shows a non-stationary time series with both a changing variance and a non-monotonically changing mean.

We separated microbial dynamics into autoregressive and non-autoregressive components by applying vector autoregressive (VAR) models, which were originally developed for econometrics [28–30]. We took this approach because we found that substantial autocorrelation persisted in most microbial time series for at least 3 days (i.e. past values of an OTU were predictive of its current value), which meant that temporally adjacent samples were not independent. VAR models are standard for analyzing stationary multivariate time series with autocorrelation, cross-correlations, and noise. Time series are considered to be stationary if they appear to be sampled from the same probability distribution through time (i.e. the mean and variance, along with the other moments of the distribution, do not change through time; Fig 1). VARs model each element as a linear function of lagged values of other elements in the time series [29]. In order to reduce the number of coefficients generated by classical VAR models (i.e. with *n* species in a VAR model with *p* lags, *n*^2^ x *p* coefficients are generated) and avoid over-fitting we apply regularized estimation, resulting in a sparse VAR (sVAR) [30, 31]. The residual variation of an sVAR model is stripped of much of its autoregressive structure, which allows for the application of standard statistical techniques that assume sample-to-sample independence. sVARs have the benefit of explicitly modeling error, unlike LV-type models [32], and are more straightforward to interpret than CCM forecasting [25].

### Multiple possible drivers of dynamics

We suggest that there are two differentiable sets of drivers generating autoregressive and non-autoregressive microbial community dynamics in the gut. The first set of drivers induce multi-day recovery processes, where the past state of the system is predictive of the future state [33]. As mentioned above, LV models are differential equations that can incorporate species-species and species-resource interactions. These models are inherently autoregressive and are the dominant workhorses of ecological time series modeling [19, 26, 34]. The second type of driver is non-autoregressive, and likely includes dietary factors and other external perturbations [10]. In this paper, we show that there are indeed two dynamic regimes: auto-regressive and non-autoregressive. These dynamic regimes appear to reflect internal and external drivers, respectively. The emerging picture of the gut microbiome shows a dynamically stable system, continually buffeted by internal and external forces and recovering back toward a conserved steady-state.

### Theoretical considerations

In this section, we directly compare our VAR modeling approach to the more common generalized Lotka Volterra family of models (gLV). gLVs are first-order differential equations that model growth rates as a non-linear function of community composition, and thus assume the existence of mechanistic coupling between variables in the system. VAR models, by contrast, assume linear dynamics, but can only be applied when the observed data are, or have been transformed such that, the time series are stationary. In both gLV and VAR models, dynamics are defined by species-species (or species-resource) interaction terms. In this study, we find that a linear VAR-based approach was sufficient to extract essential dynamics of the system without the need for a nonlinear mechanistic framework (i.e. as implemented in gLV).

gLVs have the following structure:

$$\frac{dX_{i}}{dt}=a_{i}X_{i}(t)\left(1-\frac{X_{i}(t)}{K}\right)+X_{i}(t)\sum_{j=1(j!=i)}^{n}B_{ij}X_{j}(t)$$

where *t* is time, *a*_*i*_ is the self-interaction term for organism *i*, *X*_*i*_ is the abundance of organism *i*, *B*_*ij*_ is the interaction term between organisms *i* and *j* in a community composed of *n* organisms.

Dividing by abundance and converting to difference equation form allow for gLV parameters to be solved with a system of linear equations as for a VAR(1) process [22]:

$$log(X_{i}(t))-\log\left(X_{i}(t-1)\right)=a_{i}-\frac{a_{i}}{K}*X_{i}(t-1)+\sum_{j=1(j!=i)}^{n}B_{ij}X_{j}(t-1)$$

We can now compare this linearized gLV to a corresponding VAR(1) process:

$$X_{i}(t)=q_{i}+s_{i}*X_{i}(t-1)+\sum_{j=1(j!=i)}^{n}R_{ij}X_{j}(t-1)+e_{i}\;(t)$$

where *e*_*i*_*(t)* is the error term. Thus, both gLV and VAR(1) can be solved using systems of linear equations, but the interpretation of the coefficients will be different. For instance, we can compare *a*_*i*_ (1/time) and for *q*_*i*_ (abundance), to see that VAR models directly model data on observed data, whereas gLV models assume that a more appropriate model maps observed abundance data to differenced log-transformed data.

**Fig 2:**
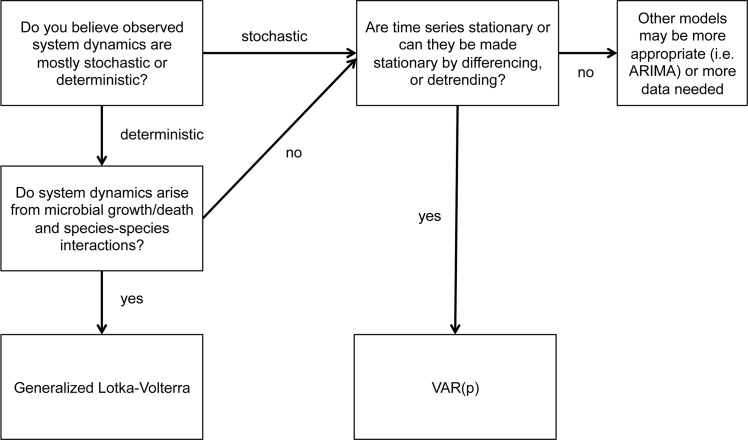
Flow chart indicating what time series modeling approach is appropriate given the structure of a data set.

Further, VAR(p) models can include an arbitrary number of time lags (i.e. autoregressive processes can extend further back in time). gLVs do not allow for explicit inclusion of historical time series data beyond one time lag. In considering the appropriate approach for analysis of microbial community time series, we created a decision tree to guide appropriate modeling strategies (Fig 2).

## Methods

### Data processing

#### Sequence data

Raw 16S (V4-V5) amplicon sequence time series data were obtained from David et al. (2014) [10] and Caporaso et al. (2011) [11]. The four data sets from these two papers are the longest, densest human gut time series currently available. Having long, dense time series is crucial for gaining an understanding of autocorrelation decay timescales and for fitting models to these large multivariate systems. 16S (V3-V5) amplicon data was also obtained from the Human Microbiome Project [1]. Raw data was quality filtered and demultiplexed using Quantitative Insights into Microbial Ecology (QIIME) default settings [35]. In order to compare across studies and reduce technical variance between studies, closed reference Operational Taxonomic Units (OTUs) were clustered at 99% identity against the Greengenes database v. 13_5 [36]. Open reference OTU picking was also run [37], in order to look for non-database OTUs that might contribute substantially to community dynamics. Time series OTU tables (i.e. *n* x *m* matrices, with *n* OTUs and *m* samples) were normalized by random sub-sampling to contain 10,000 reads per sample. Alpha and beta-diversity metrics (Shannon entropy and Jensen-Shannon divergence) were calculated with custom Python scripts using normalized OTU tables. To reduce noise from sequencing or PCR artifacts in further downstream analyses and to reduce the complexity of our models, only the top 50 most abundant OTUs or the 76 OTUs common across all time series were analyzed. The time series from donors A and B in the David et al. (2014) paper had significant diarrheal events resulting from travel abroad and food poisoning. We wanted to focus on the dynamics of normal, healthy gut communities. Thus, for donor A we focused on days 150–365 (after return from traveling abroad) and for donor B we focused on days 0–150 (prior to food poisoning event). No significant perturbations were reported in the Caporaso et al. (2011) study, so all time points were used in the analysis. 268 HMP samples were sub-sampled to contain 1,000 sequences per sample and OTUs that occurred in fewer than 10 samples were removed.

We included analyses on two non-gut time series from a marine and a freshwater ecosystem. OTU tables and metadata for these studies obtained from the Earth Microbiome Project (EMP) [38]. The EMP study numbers were 1240 (English Channel marine time series) and 1242 (Lake Mendota freshwater time series). The OTU tables and metadata files from these studies can be downloaded from FigShare: https://figshare.com/articles/Time_Series_Meta-Analysis_Files/3581616

#### Interpolation between missing time points and first-differencing

Each of our time series data sets had a few gaps (i.e. missing days). These gaps needed to be filled in order to apply our autoregressive modeling approach. Thus, missing data points were filled in using the SciPy [39] implementation of piecewise cubic interpolation (PCHIP). There are many alternative methods for interpolation that can be used (e.g. see SciPy documentation on interpolation methods: http://docs.scipy.org/doc/scipy/reference/interpolate.html) [40], but PCHIP had certain properties that were desired: abundances stay above zero; monotonicity is preserved; does not overshoot when data is not smooth [41].

OTU relative abundance matrices (*m* time points x *n* OTUs) were converted into rate matrices (*m-1* time points x *n* OTUs) by subtracting the relative abundance of each OTU at time *t* from its abundance at time *t+1* (i.e. first-differencing). The rate matrix (i.e. the first-difference or first-derivative matrix) shows the dynamics in rates between adjacent time points for each OTU in the data set.

### Data analyses

#### State clustering

In order to determine whether microbial communities clustered into discrete states (i.e. community configurations that are non-continuously distributed throughout state-space), we fit a Dirichelet multinomial mixture model (DMM) to the data [42]. The DMM clustering null model assumes that the data are pulled from the same Dirichelet multinomial distribution, and increases the number of parent distributions depending on the heterogeneity of the input data. The default Laplace method was used to penalize model complexity (http://bioconductor.org/packages/release/bioc/manuals/DirichletMultinomial/man/DirichletMultinomial.pdf) [42]. We sampled each time series at different levels of temporal resolution (i.e. every time point, every other time point, every third time point, etc.) to determine whether the number of states was influenced by over- or under-sampling of the state space. We restricted the analysis to the top 50 most abundant OTUs within each time series or the 76 abundant OTUs that occurred across all time series.

#### Abundant, conditionally rare taxa

We looked for organisms that were transiently abundant across the time series by filtering for OTUs with coefficients of bimodality > 0.8 and whose peak abundance was ≥ 10% of the total community [43]. These conditionally rare taxa (CRTs) are rare or undetected across most time points, but occasionally bloom into high-abundance (i.e. ≥ 10% of the community sequence reads).

#### Autocorrelation, cross-correlation, and stationarity

Autocorrelation decay was determined for each OTU using the autocorrelation function (i.e. acf) in the StatsModels package in Python (http://statsmodels.sourceforge.net/stable/index.html) [44]. Stationarity (i.e. whether a time series is sampled from the same statistical distribution along its entire trajectory) was assessed using the combined results of Augmented Dickey-Fuller (ADF) [45] and Kwiatkowski-Phillips-Schmidt-Shin (KPSS) tests (both TREND and LEVEL) [46]. The ADF test from the StatsModels package was used. An implementation of the KPSS test, written by Deniz Turan (http://denizstij.blogspot.co.uk/), was used (see Supplemental Materials for KPSS code).

#### Time series model

**Fig 3:**
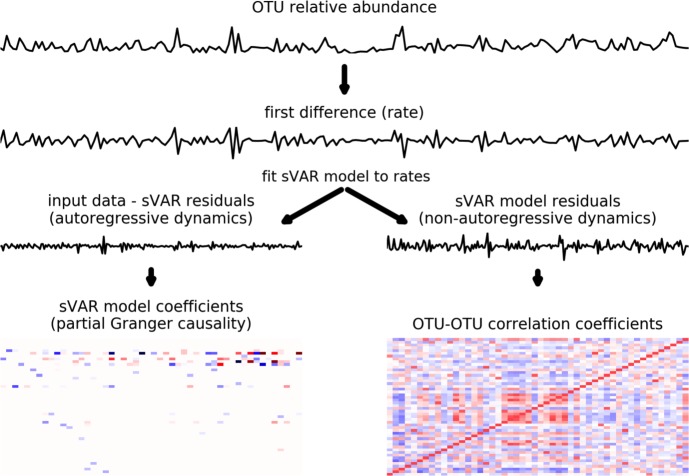
Two dynamic regimes in the gut microbiome. The top panel shows the relative abundance trace for an OTU. The second panel shows the same time series after first-differencing (i.e. taking the derivative to obtain the rate). In this data set, first-differencing ensured that each time series was stationary and appropriate for sparse vector autoregressive (sVAR) modeling. The third row of plots shows autoregressive and non-autoregressive rate dynamics, based on fitting the sVAR model. Autoregressive model coefficients (bottom left) provide information on time-lagged interactions between OTUs within the model (i.e. equivalent to partial Granger coefficients). OTU-OTU covariance (bottom right) of non-autoregressive dynamics provides information on co-fluctuating taxa.

For each time series, the top 50 most abundant OTUs were fit to a lag-3 sparse vector autoregressive model (i.e. sVAR(3), where ‘(3)’ indicates that 1–3 day lags were considered) using the sparsevar package in R v.3.2.8 (https://github.com/svazzole/sparsevar) (Fig 3) [30]. Elastic Net regularization was used for the sVAR fitting [47]. Thresholding was enforced so that very small coefficients were converted to zeros. Autocorrelation or cross-correlation structure was reduced in the sVAR(3) residuals, which allows us to more confidently apply standard statistics (i.e. methods that assume sample-to-sample independence) to the model residuals (Fig 3). Higher-order lags and rare taxa were not considered in order to minimize the number of estimated parameters. Granger causality coefficients were calculated for each non-zero sVAR coefficient [29], and significance was assessed using a chi-squared test (p < 0.05). Granger causality is based on linear time-lagged prediction: if time series A is predictive of the future values of time series B, it is said that A Granger causes B [48]. Granger causal interactions do not imply direct causality, as these associations may arise due to indirect influences.

#### Correlation and phylogenetic structure

OTU-OTU Spearman’s rho matrices were computed from the OTU matrices (i.e. relative abundances, rates, and rate residuals from the sVAR model) using Pandas [49]. Pairwise phylogenetic distances between OTU representative sequences were calculated using the dnadist function (default settings) in PHYLIP [50]. Spearman coefficients between OTUs were combined into phylogenetic distance bins, and the average coefficient for each bin was calculated.

#### Analysis code and notes

The data files and python code used to carry out the analyses presented in this manuscript, along with notes showing commands run in R, are available on FigShare: https://figshare.com/articles/Time_Series_Meta-Analysis_Files/3581616

## Results and discussion

### Community stability

Database (Greengenes) OTUs accounted for 95–99%, 93–99%, 83–97%, and 83–96% of all sequences per time point in the F4, M3, DA, and DB time series, respectively. The proportion of non-database OTUs was quite stable over time. Furthermore, the 50 most abundant OTUs in each time series were all Greengenes OTUs. Non-database OTUs tended to be low-abundance taxa.

**Fig 4:**
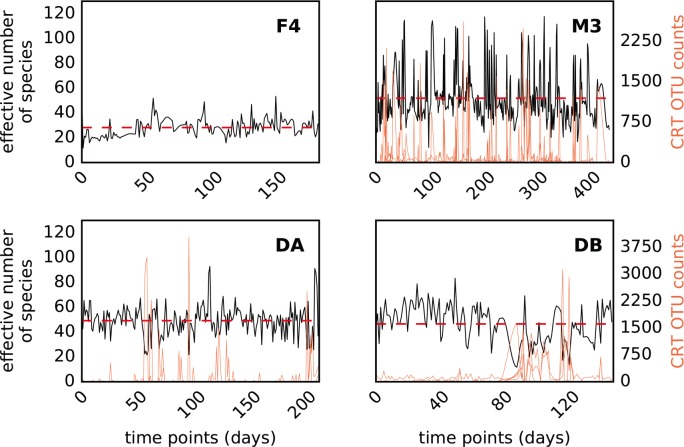
Alpha diversity is correlated with conditionally rare taxa (CRT) blooms. Black lines show the Shannon effective number of species (i.e. N_*eff*_, a measure of alpha diversity) for each time series used in this analysis. The red dashed lines show the average N_*eff*_ for each time series. The average N_*eff*_ is between 28–51 across the time series, which is high enough that compositional effects are expected to be negligible (Freidman and Alm, 2012). Major perturbations in alpha diversity are associated with CRT blooms (orange lines = sum of CRT abundances).

Microbial community alpha diversity showed stable, mean-reverting behavior across all four time series (Fig 4). The average effective number of species (*N*_*eff* =_ *e*^*[Shannon diversity]*^) was between 28–50 for each time series, which indicated that compositional effects (i.e. spurious correlations caused by non-independence between relative abundances) were not a major concern in this analysis (Fig 4) [51]. Friedman and Alm (2012) found that, in simulated data, for a *N*_*eff*_ of ~30 or more, conventional statistics gave the same result as their compositionally aware method. We found that large deviations in alpha diversity were strongly correlated with a set of conditionally rare taxa (CRTs) that occasionally bloomed to as much as ~30% of the community (dominant taxa at steady-state were usually between 10–20% of the community), but were usually found at very low abundances (Fig 4). In order to test whether CRT blooms drove significant compositional effects in the correlation structure of our time series, we calculated Spearman’s correlations between the 100 most abundant taxa in the M3, DA, and DB time series with and without the CRT time points (i.e. time points where CRTs rose above 10% relative abundance were removed; S1 Fig). Overall, we saw no compositional effects due to CRT blooms (S1 Fig). A recent meta-analysis by Shade and Gilbert (2015) found that CRTs are responsible for a significant fraction of overall community dynamics in many different ecosystems [43]. Abundant gut CRTs—*Prevotella*, *Bacteroides fragilis*, *Akkermansia muciniphila*, *Lachnospiraceae*, *Enterobacteriaceae* and *Haemophilus parainfluenzae*—were present in M3, DA, and DB time series (S1 Table; Fig 4). Abundant CRTs (i.e. peak abundance ≥ 10% of sequence reads) were not identified in the F4 time series, consistent with its relatively stable alpha-diversity trace (Fig 4). However, there were several *Enterobacteriaceae* blooms in the F4 time series that fell beneath our abundance threshold. The M3 time series was an outlier, with much more frequent CRT blooms than the other time series. These bloom events likely represent opportunistic or pathogenic organisms that are either the cause or symptom of a disruption in the normal steady-state gut environment. Many CRT OTUs are facultative anaerobes or aerotolerant taxa (e.g. *Enterobacteriaceae* OTUs and other Proteobacteria or OTUs in the *B*. *fragilis* group, like *B*. *uniformis*, *B*. *ovatus*), which are probably responding to changing redox potential in the gut following some disturbance (e.g. inflammation) [52].

**Fig 5:**
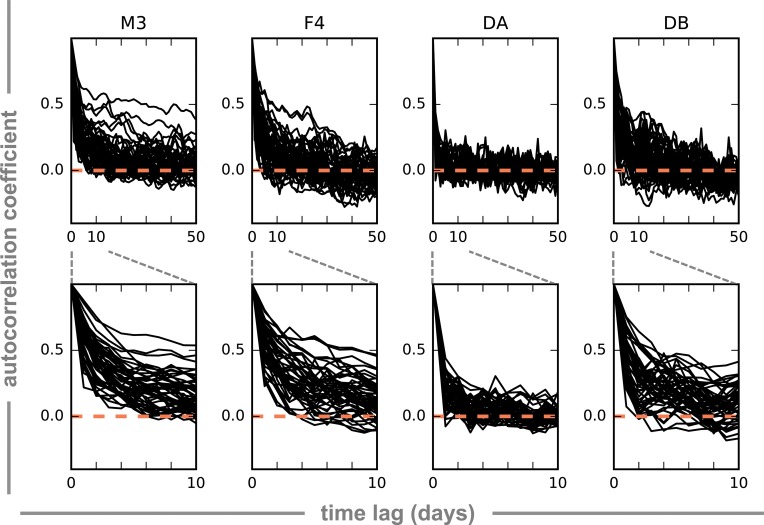
Autocorrelation decay plots for the 50 most abundant OTUs in each time series. Each black line represents the autocorrelation for a single OTU over different time lags (in days). The top row of plots shows autocorrelation (y-axis) for up to 50-day lags (x-axis). Orange dashed lines indicate an autocorrelation coefficient of 0.0. Dashed grey lines show the zoom-in region from the upper row of plots presented in the second row of plots (lags up to 10 days).

After first-differencing (i.e. differencing OTU abundances at adjacent time points), abundant OTUs showed completely stationary dynamics, implying that the mean, variance, and autocorrelation structure do not change over time (ADF and KPSS tests; Table 1). Both ADF and KPSS Level tests (Table 1) assess whether or not a process has a unit root, which implies that the mean changes through time and that the system does not recover to the mean trend in the presence of a shock (i.e. the process is integrated). The KPSS Trend test (Table 1) assesses whether or not a process is trend-stationary, which implies a mean trend with stationary error that is capable of recovering to the trend line following a shock. Non-stationary OTUs are likely not stable members of the steady-state community. Prior to first-differencing, most OTU trajectories showed ADF-stationary dynamics (Table 1). There was no apparent enrichment for particular taxonomic groups among non-stationary OTUs. In addition to OTU abundance trajectories being largely stationary, we also saw a range of autocorrelation decay curves for the top 50 most abundant OTUs (Fig 5). Some OTUs showed strong, persistent autocorrelation, while others did not (Fig 5). The amount of autocorrelation decay also varied across time series. In particular, the DA time series showed much less autocorrelation than the other three time series (Fig 5).

**Table 1:** Percentage of abundant OTUs (50 most abundant OTUs from each time series) that show stationary dynamics before (raw) and after (delta) first-differencing. Three tests of stationarity were used: the augmented Dickey-Fuller (ADF) test, the Kwiatkowski–Phillips–Schmidt–Shin (KPSS) trend test, and the KPSS level test.

	ADF	KPSS Trend	KPSS Level
**M3 raw**	92.0	26.0	34.0
**M3 delta**	100.0	100.0	100.0
**F4 raw**	80.0	44.0	40.0
**F4 delta**	100.0	100.0	100.0
**DA raw**	100.0	48.0	90.0
**DA delta**	100.0	100.0	100.0
**DB raw**	88.0	22.0	44.0
**DB delta**	100.0	100.0	100.0

**Fig 6:**
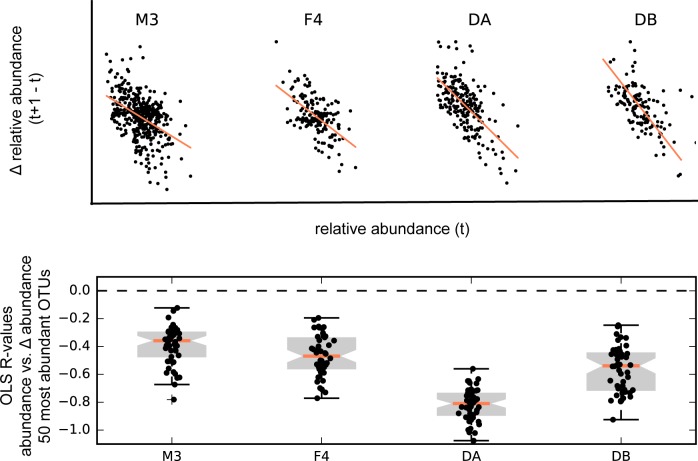
OTUs in the time series data show a propensity for mean-reversion. The top row of plots shows the change in relative abundance from time t to time t+1 (y-axis) vs. the relative abundance (x-axis) for OTU 850870 (Greengenes ID; *Bacteroides spp*.; the most abundant OTU in M3, F4 and DB time series and 2^nd^ most abundant OTU in DA time series). The bottom plot summarized the ordinary least-squares (OLS) regression coefficient (R) for the top 50 most abundant OTUs from each data set. In general, there is a negative association between an OTU’s change in abundance and its current abundance level, suggesting that OTUs have fixed carrying capacities. The average magnitude of the regression coefficient appears to be an indicator of the returning force. These results are consistent with stationarity.

Stationarity implies that there is a restoring force on an OTU's abundance over time, so that it returns to a mean value after a perturbation (i.e. a steady-state population size, or ‘carrying capacity’). Indeed, we found a significant negative correlation between the change in OTU abundances between time *t* and time *t+1* and the abundances of OTUs at time *t* (Fig 6). Thus, by converting abundance dynamics to rate dynamics, we can achieve stationarity across all abundant time series in the system and preserve correlation structure between OTUs, as long as we exclude non-stationary windows that contain large disturbances (e.g. food poisoning in the DB time series on day 150). Furthermore, the strong correlation between rates and OTU abundances at the prior time step indicates that much of the variance in these data is linear (Fig 6). The fact that these rate dynamics can be modeled as linear, stationary processes opens up a wide array of time series models to gut data sets.

### Discrete vs. continuous dynamics in the gut microbiome

Prior work has argued for the existence of discrete gut community configurations across humans, termed ‘enterotypes’, that may be associated with health and disease [12, 14]. However, longitudinal data has provided evidence that these enterotypes may arise from undersampling individuals through time. For example, the M3 time series is known to moved fluidly through all three putative enterotypes from Arumugam et al. (2010) during the course of a year [13], which suggests that gut communities vary along a continuum. We expanded upon this analysis using an improved Dirichlet multinomial mixture model (DMM) clustering method [14, 42], which assesses whether samples appear to be pulled from a common Dirichlet multinomial distribution. In general, clustering methodologies are plagued by over- and under-sampling issues. In a time series, samples that are taken close together in time are likely to be similar to one another (i.e. they are autocorrelated). If the community is sampled densely enough as it moves through state space, then packets of samples that happen to be temporally adjacent may be grouped into pseudo-clusters (i.e. over-sampling). On the other hand, if only a handful of samples are taken from an individual, then outlier points may give rise to pseudo-clusters because the state space was not sufficiently sampled (i.e. under-sampling). If there are no discrete states to be found, then the number of states should decay smoothly to 1 as the sampling sparcity is increased. However, if discrete states do exist, then the number of states should reach a plateau that is stable across a range of sampling densities.

**Fig 7:**
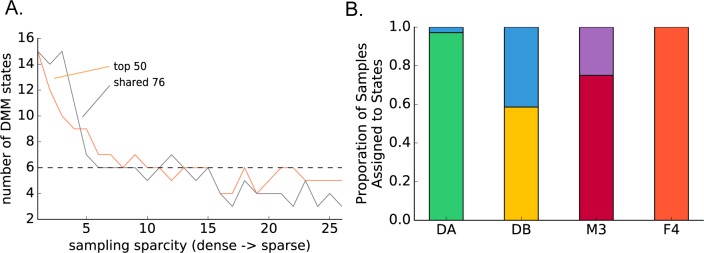
(A) shows decay in the number of Dirichelet multinomial mixture model (DMM) states as the sparcity of the time series is increased for the top 50 most abundant OTUs from each time series and for the 76 abundant OTUs shared across all four time series. The plateau is at 6 states for both methods (grey dashed line). (B) shows state classifications for samples taken from all four individuals (top 50 OTUs from each time series sampled at every 11^th^ time point). States are largely unique to individuals (i.e. colors indicate unique states), with the exception of a single time point in the DA series that was assigned to a state (blue) that is dominant in DB.

We aimed to address whether gut communities can be grouped into discrete states and whether these states are shared across individuals given a range of intra-individual sampling effort. DMM states were fit to the full-length time series, which included the food poisoning event in the DB time series. Independent of our OTU filtering method (50 most abundant OTUs in each time series, or 76 abundant OTUs shared across all time series), we found that the number of DMM states decayed rapidly from 15 to a plateau of 6 (Fig 7A). These states were almost entirely unique to individuals (Fig 7B). There was only one case where a sample from DA was assigned to a state largely associated with DB. In addition, only DB and M3 time series harbored multiple states, which is consistent with major perturbations in the gut community within these time series (i.e. food poisoning and frequent CRT blooms, respectively). Overall, barring major perturbations, we conclude that individual humans—given sufficient sampling density—can be distinguished by unique Dirichelet multinomial distributions (Fig 7). Because almost every time point from a subject’s time series is assigned to the same one or two DMM states, continuous methods are necessary for exploring the dynamics of microbial communities within an individual. We believe this result will hold as larger numbers of long-term human gut time series become available.

**Fig 8:**
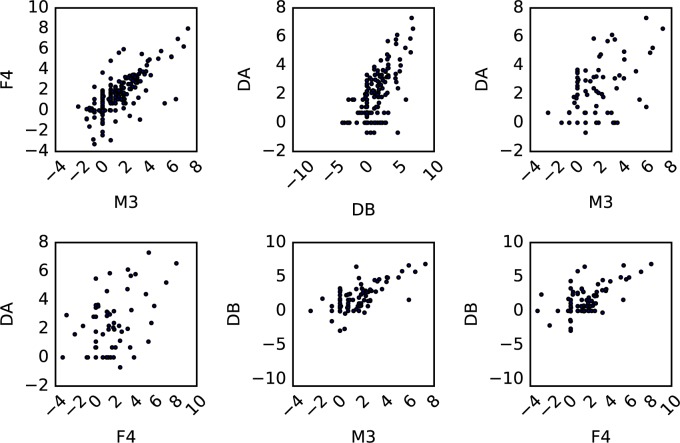
Scatter plots of the log median abundances of shared 99% OTUs across time series. Taxa that are present across time series show a positive correlation in their median abundances.

Despite the clustering of individuals into unique states, we identified 956 OTUs that were present across all four individuals. These organisms tended to be found at similar median abundances across individuals (Fig 8). In addition, these shared OTUs made up 70–80% of the sequences in each data set (Table 2). Furthermore, a single OTU (from the genus *Bacteroides*; Greengenes ID 850870) was the most abundant taxon in M3, F4 and DB, and was the second most abundant organism in DA (Fig 6). Thus, despite the fact that most OTUs appear to be unique to an individual’s gut, there is a set of abundant core taxa that are present at similar abundances across people. It is unlikely that compositional effects are responsible for the existence of these carrying capacities, due to the fact that there is a wide-range in of abundances at which OTUs appear to persist (Fig 8). With similar carrying capacities not only within but also across individuals, these core OTUs likely occupy similar metabolic niches across humans.

**Table 2:** Number of OTUs shared across the time series (M3, F4, DA, and DB) and human microbiome project (HMP) data sets. The 956 OTUs shared across all four time series make up the following percentages of total abundances in each time series: M3 = 69.9%; F4 = 77.2%; DA = 76.9%; and DB = 79.9%. The percent of total community abundance made up by the 308 OTUs shared across all data sets (including HMP) are as follows: M3 = 61.1%; F4 = 70.0%; DA = 61.7%; DB = 59.1%; and HMP = 39.3%.

	M3	F4	DA	DB	HMP
**M3**	7260				
**F4**	3436	4334			
**DA**	2109	1431	4839		
**DB**	1796	1234	2804	3910	
**HMP**	1214	820	855	788	3293

*shared across 4 time series: 956

*shared across all data sets: 308

### Sparse vector autoregression (sVAR) and granger causality

We employ an analytical approach based on continuous, multivariate, linear, autoregressive modeling tools developed for econometrics to pull apart two independent dynamic regimes in the human gut microbiome (Fig 3). Each regime, autoregressive and non-autoregressive, tells a unique story about the gut ecosystem.

**Fig 9:**
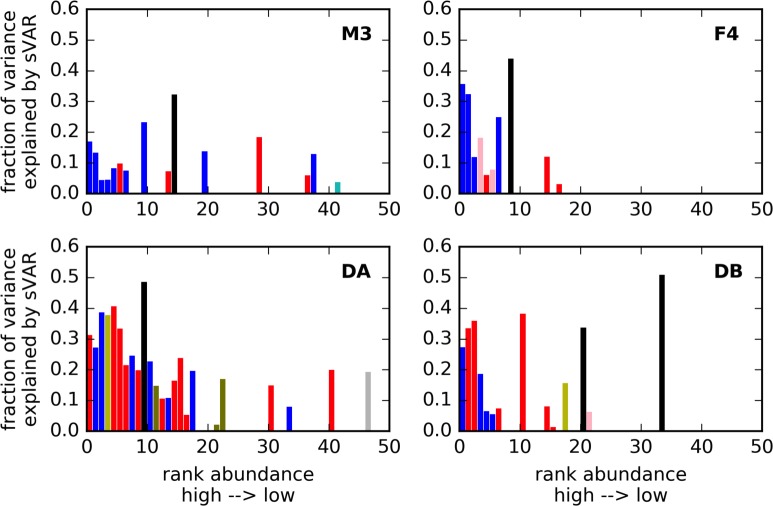
Amount of variance explained by sVAR(3) model for top 50 OTUs in each time series. Bars are colored by order: blue = *Bacteroidales*, red = *Clostridiales*, black = *Enterobacteriales*, pink = *Verrucomicrobiales*, yellow = *Erysipelotrichales*, olive = *Bifidobacteriales*, silver = *Pasteurellales*, cyan = *Synergistales*.

OTU abundance trajectories showed fairly strong autocorrelation structure (Figs 5 and S2), although there was no evidence for auto-covariance in a limited set of dietary metadata from the DB time series (S3 Fig). The autocorrelation decay curves showed that most of the autocorrelation was gone after a lag of 3 or 4 days in the abundance data, and most of the autocorrelation was gone from the differenced time series after 1 or 2 days (S2 Fig). Thus, we chose to fit a lag-3 sparse vector autoregressive model to all the data to account for this autoregressive signal. sVAR(3) models produced residuals with reduced autocorrelation structure (S2 Fig). The sVAR models (i.e. the linear autoregressive components of the variance) accounted for a minority of the total community variance (0–50% for any given OTU; Fig 9). The set of OTUs with strong autoregressive signals were phylogenetically heterogeneous (S4 Fig). OTUs from the *Enterobacteriaceae* family tended to have larger amounts of their variance explained by the sVAR than other taxa (Fig 9). These *Enterobacteriaceae* OTUs were also often identified as CRTs, blooming periodically from low to high abundance. The most abundant taxa also tended to show more autoregressive structure than lower-abundance taxa (Fig 9).

**Fig 10:**
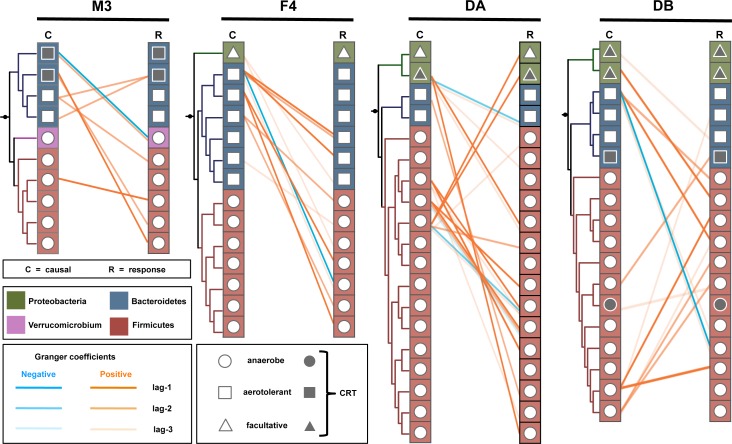
Granger causal networks for each time series. Trees show phylogenetic relationships between taxa. Edges are interactions identified in the sVAR(3) model that also show significant Granger causality (p < 0.05). Symbols indicate oxygen tolerance and growth traits. CRT stands for ‘conditionally rare taxon’, which is defined by a coefficient of bimodality > 0.8 and a maximum abundance of > 10% of the community. CRTs are organisms that are usually rare, but occasionally bloom to very high abundance. CRTs tend to be facultative aerobes, or aerotolerant taxa.

Many sVAR coefficients showed significant Granger causal associations (i.e. the past abundances of one OTU predict the future abundances of another OTU; Fig 10; Chi-Squared test, p < 0.05). The M3 time series had the sparsest Granger network, with no significant relationships for 3-day lags. This lack of significant Granger relationships at 3-day lags may be due to the higher frequency of CRT blooms in that time series, which may have continually disrupted community recovery and obscured successional trends. A *B*. *fragilis* OTU had the largest number of connections in the M3 Granger network. *B*. *fragilis*, *B*. *ovatus*, and *Enterobacteraceae* OTUs had the largest numbers of connections in the F4 Granger network. In the DA and DB Granger networks, *F*. *prausnitzii* OTUs had the largest number of significant Granger interactions (Fig 10). Overall, *Bacteroides*, *Faecalibacterium*, and *Enterobacteriaceae* OTUs were prevalent in all the Granger networks (Fig 10). In each Granger network, there were several Granger-causal OTUs that influence multiple downstream responder OTUs, but there were only a few responder OTUs (e.g. *B*. *fragilis*, in the F4 time series) that integrate multiple upstream Granger signals. This pattern is consistent with cascading dynamics that result from perturbing a highly connected/central node in a network with an external shock [53]. In general, facultative aerobes (e.g. *Enterobacteraceae* OTUs) and aerotolerant taxa (e.g. *Bacteroides*) were more likely to Granger-cause obligate anaerobes (e.g. most Firmicutes OTUs) than the reverse (Fig 10). These intrinsic dynamics suggest a successional process that might follow a spike in luminal oxygen levels [52].

### Non-autoregressive dynamics contain phylogenetic structure

**Fig 11:**
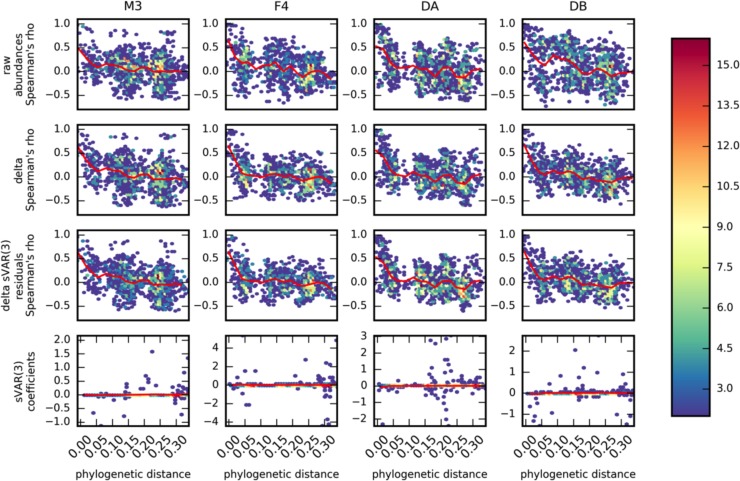
Phylogenetic relatedness corresponds to similarity in dynamics for top 50 most abundant OTUs. Each plot is a heatmap showing the density of pairwise OTU-OTU correlation or sVAR coefficients (y-axes) vs. pairwise phylogenetic distances (x-axes). The red lines show the mean coefficient along phylogenetic distance windows. Top row shows Spearman correlations calculated based on raw count data. Second row shows Spearman correlations calculated based on the first-difference (delta) of the count data. The third row shows the Spearman correlations performed on the residuals of a sVAR(3) model fit to the deltas. The bottom row shows the sVAR(3) coefficients. The heatmap colors denote the density of OTU-OTU pairs at a given hexagonal pixel on the plot.

sVAR(3) residuals showed reduced autocovariance and could thus be more appropriately analyzed using standard statistical methods that assume independence. Closely related gut bacteria were generally positively correlated with one another, but this coherence decayed rapidly with phylogenetic distance (Fig 11). There was a significant anti-correlation between OTU-OTU phylogenetic distance and OTU-OTU correlations in abundances and abundance rates through time (i.e. highly related taxa tended to be positively correlated; Spearman’s p < 0.001). This phylogenetic coherence is conserved in the sVAR(3) residuals, but is completely absent in the sVAR(3) coefficients (Fig 11). Moreover, although we did not identify strong correlations between the set of dietary variables measured for the DB time series and the DB gut community, these dietary variables showed little-to-no autocorrelation structure (S3 Fig; and see Additional File A8 in David et al., 2014) [10]. Furthermore, we fit an sVAR(3) model to the original 97% OTUs from the DA time series paper and found that none of the OTUs that had previously been shown to correlate with dietary variables had any autoregressive signal (i.e. sVAR coefficients = 0; S1 File). We suggest that unmeasured dietary variables and host behavior/physiology are the non-autoregressive drivers responsible for the pronounced phylogenetic signal in OTU-OTU residual correlation structure (i.e. related taxa are positively correlated because they share a similar host/dietary niche, which fluctuates stochastically in time). In addition to phylogenetic coherence in the correlation structure within a gut time series, we find the same phylogenetic coherence in the correlations between OTU abundances across people from the HMP gut data set (S5 Fig). Thus, microbial phylogeny is strongly coupled to host niche in the human gut. In order to assess the generality of this relationship between phylogeny and correlation, we analyzed two time series from the English Channel and from Lake Mendota (marine and freshwater environments, respectively). We found the same pattern in these non-host associated environments, suggesting that niche filtering is a strong driver of dynamics across different ecosystems (S6 and S7 Figs).

### Conclusion

Despite distinct differences in community composition across humans, the dynamics of the gut microbiota are stable and highly conserved. Dominant microbes in the gut appear to have fixed carrying capacities (i.e. their dynamics are stationary), which opens the door to many classical time series modeling approaches. Significant time-lagged interactions between OTUs often include opportunistic, facultative anaerobic organisms like *Enterobacteraceae*, and obligate anaerobes like *F*. *prausnitzii* [54, 55]. These autoregressive interactions appear to be due to succession and recovery of the gut community from CRT blooms, which may result from a disruption in the luminal redox balance [52]. The largest component of community variance is non-autoregressive and appears to be driven by non-autoregressive environmental forces, like pH [56] or fiber intake [10]. Unlike the autoregressive dynamics, these non-autoregressive dynamics carry a strong phylogenetic signal, indicative of niche filtering. Our results, based on a limited number of individuals, paint a coherent picture of the gut ecosystem and the major forces underlying its structure and stability, with two distinct dynamic regimes: one driven by external factors (e.g. diet) and the other by internal autoregressive processes (e.g. recovery following a disturbance). Moving forward, it will be important to collect more time series data, from both healthy and diseased individuals, to determine how general these dynamics are and whether or not they are observed in dysbiotic gut communities.

## Supporting information

S1 Table Abundant CRTs (max abundance ≥ 10%; coefficient of bimodality ≥ 0.8) across the four time series. (DOCX)

S1 Fig OTU-OTU correlation coefficients (Spearman’s rho) of first-differenced data are not strongly impacted by CRT blooms in the DA, DB, and M3 time series (i.e. time series where CRTs rise above 10% relative abundance). The x-axis shows the correlation coefficients for all time points and the y-axis shows the coefficients calculated with CRT time points removed. Red lines show linear regression fits (R^2^ > 0.9 across all three time series).
(TIF)

S2 Fig Autocorrelation structure in four long-term gut time series. First-differencing (i.e. calculating the rate, or delta) and subsequent sparse VAR (sVAR) modeling removes most of the autocorrelation from the data. Most autocorrelations decay to zero within 2–3 days (i.e. for the raw abundance counts), so a maximum time lag of 3 days was chosen for sVAR fitting.
(TIF)

S3 Fig Autocorrelaion decay for ten quantitative dietary variables (e.g. carbohydrate intake, fiber intake, fat intake, etc.) from the DB time series. (TIF)

S4 Fig The top 50 most abundant OTUs in each time series plotted on phylogenetic trees. Black circles denote amount of variance explained by an sVAR(3) model. Larger circles indicate OTUs with a larger autoregressive component to their variance. Tips of trees are labeled with the Family-level taxonomic annotation for each OTU.
(TIF)

S5 Fig Hexagonal 2-D heatmap showing OTU-OTU Spearman correlation coefficients (y-axis) vs. OTU-OTU phylogenetic distances (x-axis) for the 50 most abundant taxa in the Human Microbiome Project (HMP) gut data set. The heatmap colors denote the density of OTU-OTU pairs at a given hexagonal pixel on the plot.
(TIF)

S6 Fig Hexagonal 2-D heatmap showing OTU-OTU Spearman correlation coefficients (y-axis) vs. OTU-OTU phylogenetic distances (x-axis) for the English Channel marine time series data (Earth Microbiome Project Study #1240). The heatmap colors denote the density of OTU-OTU pairs at a given hexagonal pixel on the plot.
(TIF)

S7 Fig Hexagonal 2-D heatmap showing OTU-OTU Spearman correlation coefficients (y-axis) vs. OTU-OTU phylogenetic distances (x-axis) for the Lake Mendota freshwater time series data (Earth Microbiome Project Study #1242). The heatmap colors denote the density of OTU-OTU pairs at a given hexagonal pixel on the plot.
(TIF)

S1 File Percent of variance explained by an sVAR(3) model for the original 97% OTUs from the DA time series paper. OTUs that had previously been shown to correlate with dietary variables (highlighted in yellow) had no autoregressive signal (i.e. sVAR coefficients = 0).
(XLSX)
