# Supplementary material for: Two dynamic regimes in the human gut microbiome
Source: PLoS Comput Biol. 2017 Feb 21;13(2):e1005364. doi: 10.1371/journal.pcbi.1005364 (PMC5340412; doi:10.1371/journal.pcbi.1005364)
Supplement: S1 Table — (DOCX) [file pcbi.1005364.s001.docx]

**Table S1.** Abundant CRTs (max abundance ≥ 10%; coefficient of bimodality ≥ 0.8) across the four time series.

|  | number of abundant CRTs | average peak CRT relative abundance |
| --- | --- | --- |
| M3 | 3 | 0.1587 |
| F4 | NA | NA |
| DA | 2 | 0.2544 |
| DB | 5 | 0.1731 |
