# Supplementary figures and images for: Two dynamic regimes in the human gut microbiome
Source: PLoS Comput Biol. 2017 Feb 21;13(2):e1005364. doi: 10.1371/journal.pcbi.1005364 (PMC5340412; doi:10.1371/journal.pcbi.1005364)

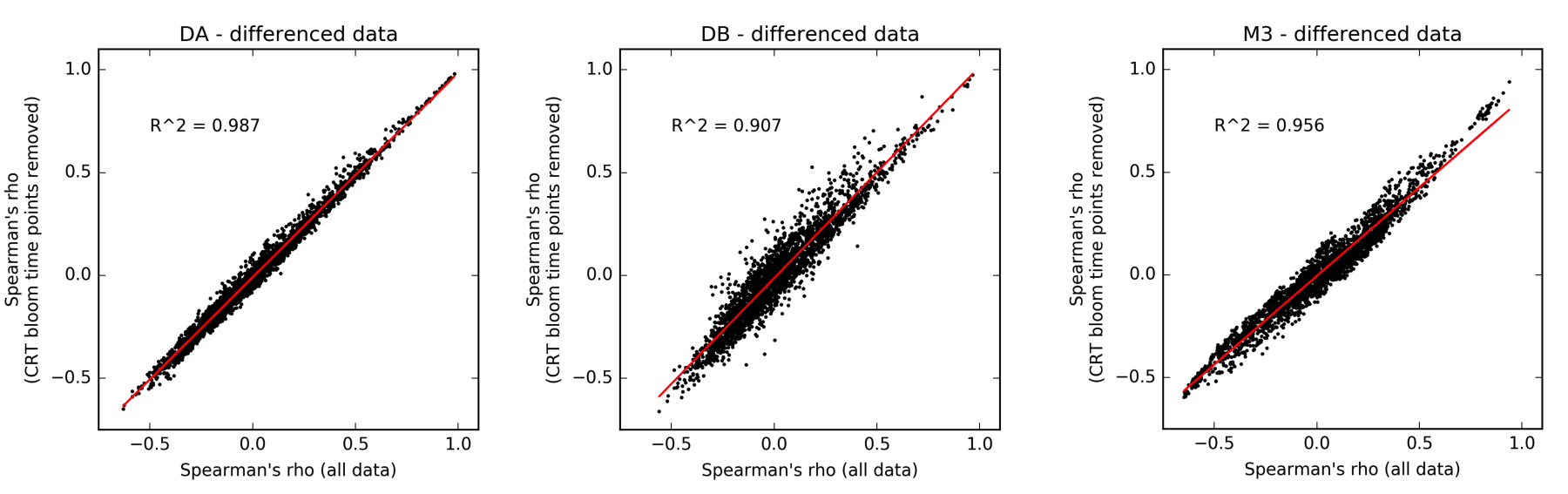

Supplement: S1 Fig — The x-axis shows the correlation coefficients for all time points and the y-axis shows the coefficients calculated with CRT time points removed. Red lines show linear regression fits (R2 > 0.9 across all three time series). (TIF) [file pcbi.1005364.s002.tif]

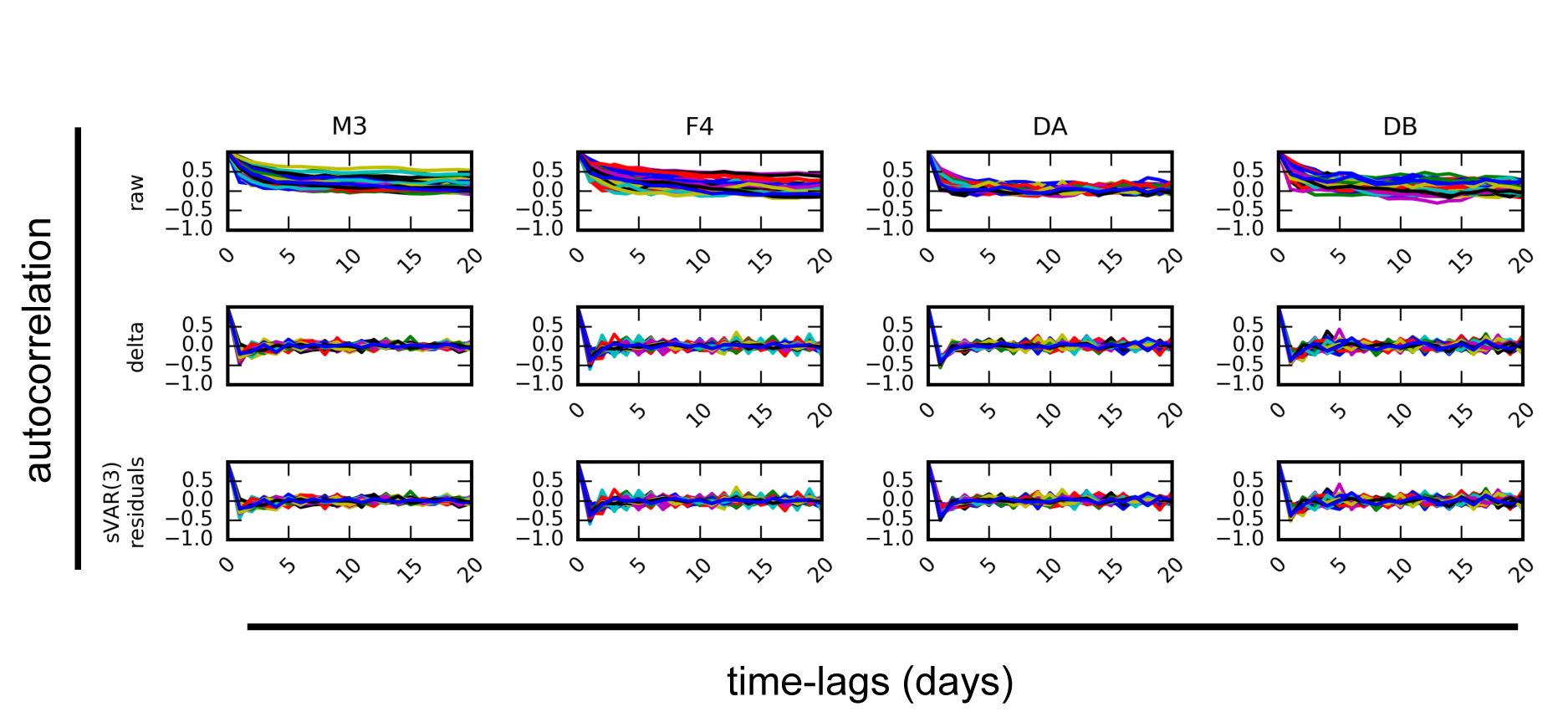

Supplement: S2 Fig — First-differencing (i.e. calculating the rate, or delta) and subsequent sparse VAR (sVAR) modeling removes most of the autocorrelation from the data. Most autocorrelations decay to zero within 2–3 days (i.e. for the raw abundance counts), so a maximum time lag of 3 days was chosen for sVAR fitting. (TIF) [file pcbi.1005364.s003.tif]

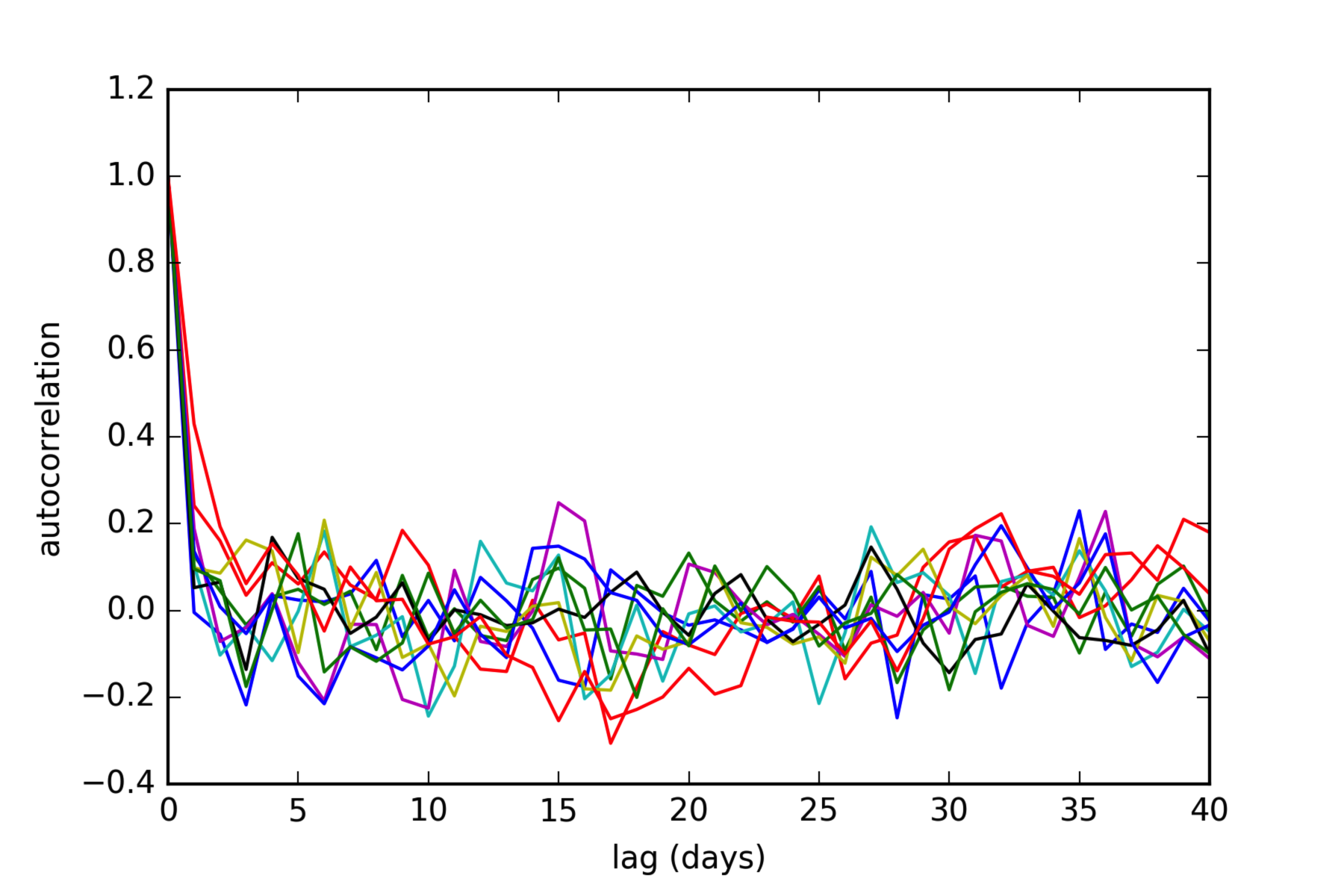

Supplement: S3 Fig — (TIF) [file pcbi.1005364.s004.tif]

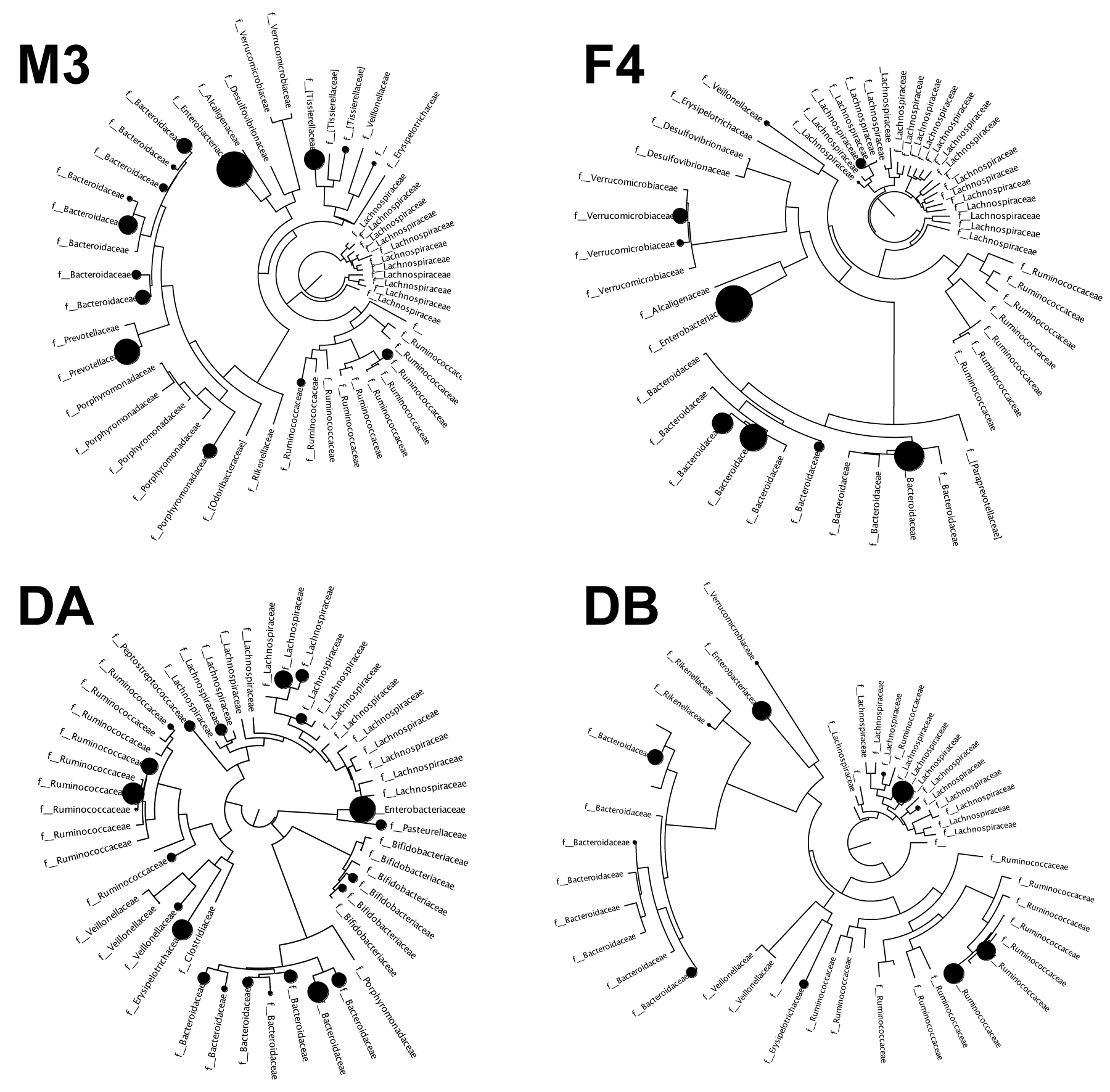

Supplement: S4 Fig — Black circles denote amount of variance explained by an sVAR(3) model. Larger circles indicate OTUs with a larger autoregressive component to their variance. Tips of trees are labeled with the Family-level taxonomic annotation for each OTU. (TIF) [file pcbi.1005364.s005.tif]

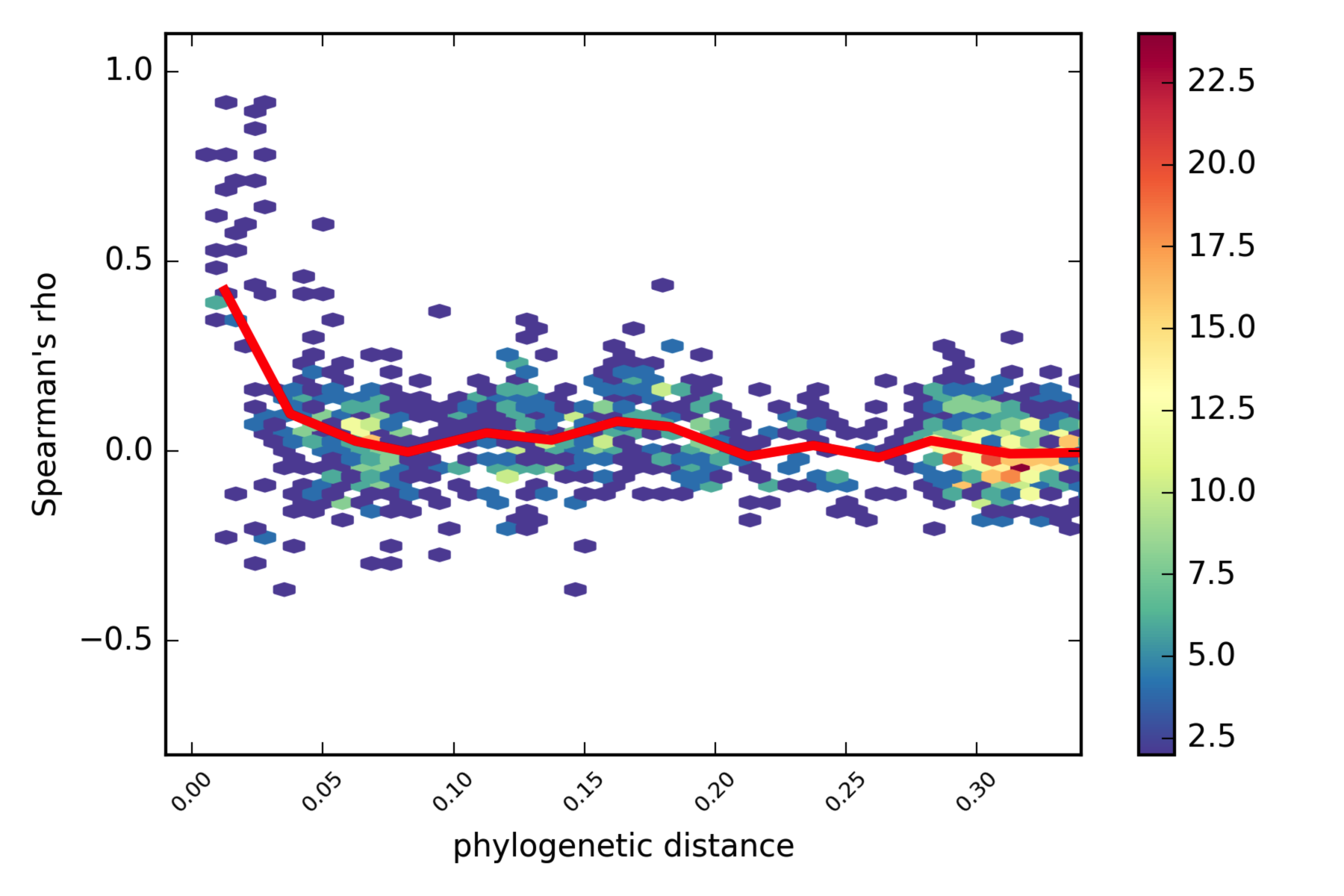

Supplement: S5 Fig — The heatmap colors denote the density of OTU-OTU pairs at a given hexagonal pixel on the plot. (TIF) [file pcbi.1005364.s006.tif]

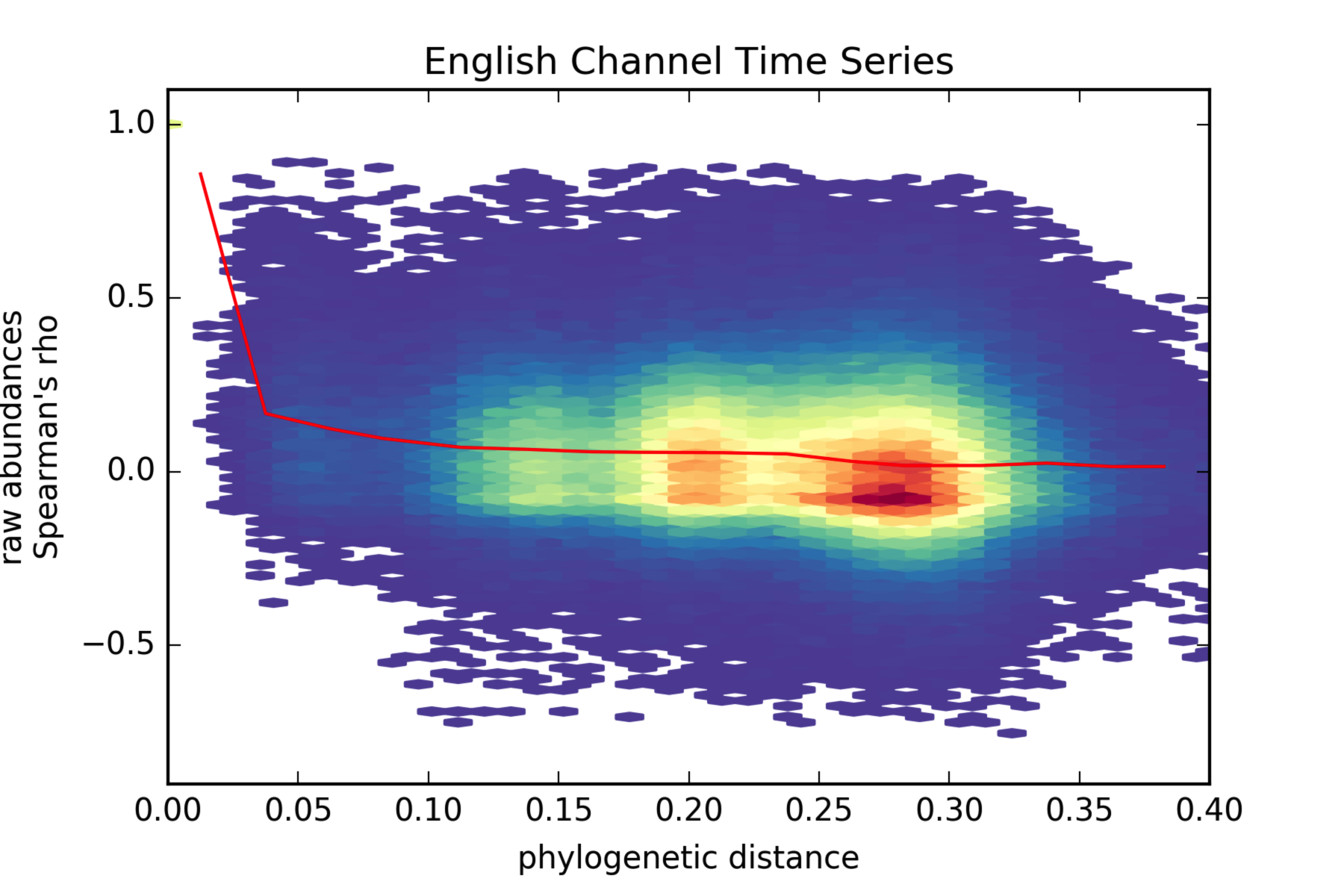

Supplement: S6 Fig — The heatmap colors denote the density of OTU-OTU pairs at a given hexagonal pixel on the plot. (TIF) [file pcbi.1005364.s007.tif]

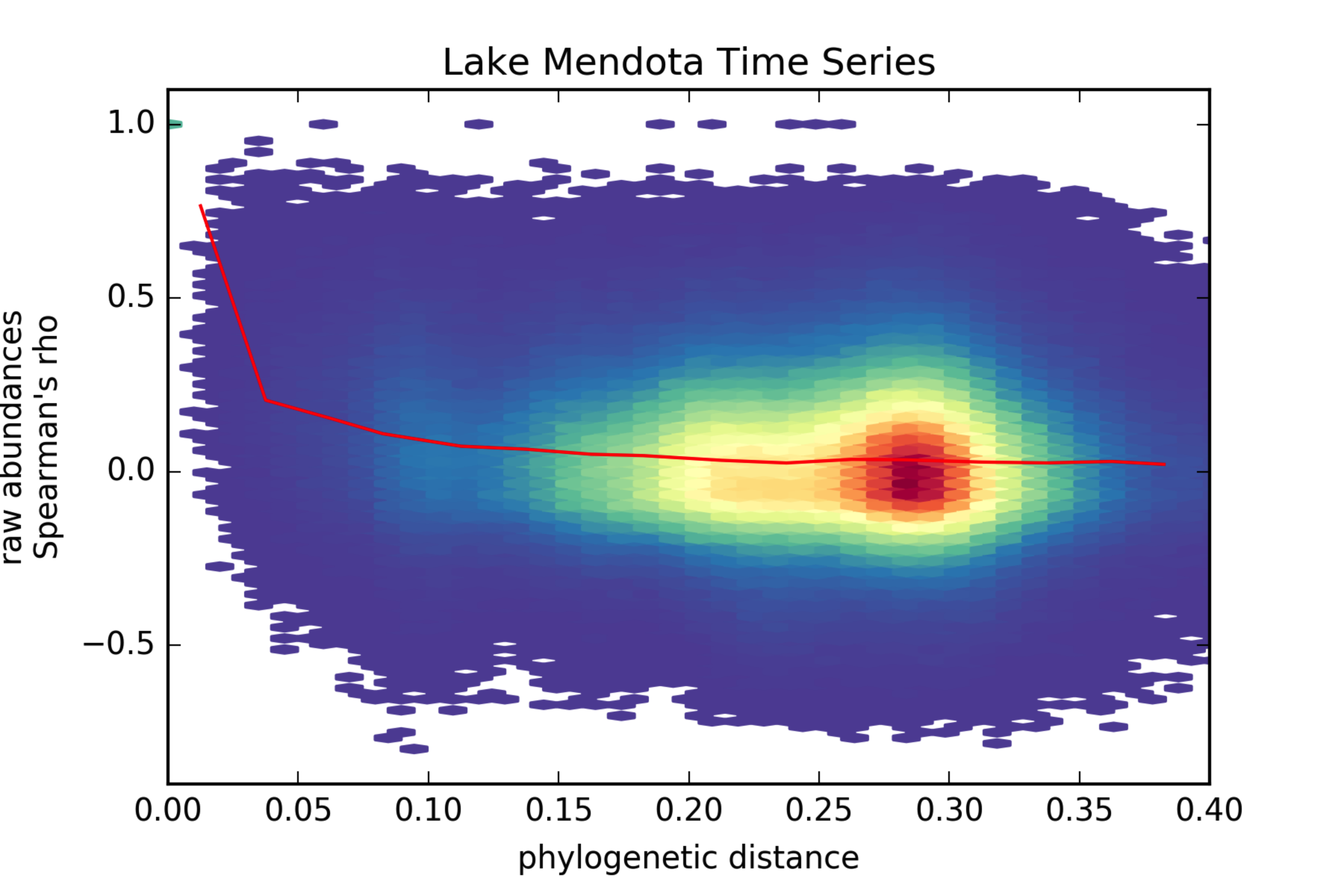

Supplement: S7 Fig — The heatmap colors denote the density of OTU-OTU pairs at a given hexagonal pixel on the plot. (TIF) [file pcbi.1005364.s008.tif]
